# Patent Foramen Ovale Complicated With Renal Infarction and Pulmonary Embolism: A Case Report With Literature Review

**DOI:** 10.7759/cureus.35433

**Published:** 2023-02-24

**Authors:** Yasuaki Iizuka, Tomoya Tsuchida, Kohei Ashikaga, Kenya Ie, Takuya Matsuda, Kosuke Ishizuka, Chiaki Okuse, Takahide Matsuda, Yoshiyuki Ohira

**Affiliations:** 1 Division of General Internal Medicine, Department of Internal Medicine, St. Marianna University School of Medicine, Kawasaki, JPN; 2 Department of Sports Medicine, St. Marianna University School of Medicine, Kawasaki, JPN; 3 Division of Nephrology and Hypertension, St. Marianna University School of Medicine, Kawasaki, JPN

**Keywords:** transesophageal echocardiography, paradoxical embolism, pulmonary embolism, renal infarction, patent foramen ovale (pfo)

## Abstract

A 48-year-old man visited our emergency room after experiencing sudden left back pain, diaphoresis, and nausea. The patient underwent physical and laboratory examinations. Physical examination revealed tenderness in the left costovertebral angle. Laboratory examination revealed a slight elevation in D-dimer levels. Contrast-enhanced computed tomography revealed a bilateral pulmonary embolism and left renal infarction. Back pain was resolved following anticoagulation therapy with heparin. Transesophageal echocardiography revealed a patent foramen ovale (PFO). The patient was discharged on an anticoagulant, apixaban. Identifying the cause of paradoxical embolisms, such as an atrial septal defect or PFO, in cases with an arterial embolism in a young patient with no underlying disease is important.

## Introduction

A patent foramen ovale (PFO) is a congenital cardiac lesion. The foramen ovale usually closes a few months after birth; however, in a PFO, it remains open even in adulthood. A PFO is found in approximately 25% of adults [1]. Although most PFO patients are asymptomatic, a paradoxical embolism is a notable complication. A paradoxical embolism is a condition in which the embolic materials cause emboli from the venous circulation to the arterial circulation through the right-left shunt, like in cases of a PFO, atrial septal defect (ASD), or ventricular septal defect [2].

While cerebral embolisms due to paradoxical embolisms are reported to account for approximately half of the cryptogenic ischemic stroke cases [3], paradoxical renal embolisms accompanied by a pulmonary embolism are rare. Herein, we report a rare case of a PFO complicated by a renal infarction and pulmonary embolism.

## Case presentation

A 48-year-old man visited the emergency room in the early morning with left back pain that had persisted for four hours, accompanied by diaphoresis and nausea. He had returned to Japan from India one day before the hospital visit. He had no relevant medical history or health check abnormality and was not taking any medication. He occasionally drank alcohol and never smoked. No chest or shifting pain was reported. Both urinalysis and non-contrast CT of the abdomen revealed no abnormalities. We believed that any ureteral stones would have passed before the CT test, and the patient was judged to have residual pain after ureteral stone drainage. The patient was treated with a 1000 mg intravenous infusion of acetaminophen. However, his symptoms did not improve, and he visited our outpatient department during the day.

The patient’s vital signs were stable. Physical examination revealed tenderness at the costovertebral angle on the left side. Cardiac and respiratory sounds were normal. An electrocardiogram showed a heart rate of 74 beats/min and a sinus rhythm. Blood tests showed mild elevation of D-dimer levels (Table 1).

**Table 1:** Laboratory findings

	Value	Unit	Normal value
Leucocyte	8400	/μL	3300-8600
Hemoglobin	14.4	g/dL	13.7-16.8
Platelet	27.1	10⁴/μL	15.8-34.8
Total bilirubin	1.4	mg/dL	0.4-1.5
Aspartate aminotransferase	31	U/L	13-30
Alanine aminotransferase	30	U/L	10-42
Lactate dehydrogenase	276	U/L	124-222
Alkaline phosphatase	238	U/L	106-322
γ-Glutamyl transpeptidase	38	U/L	13-64
Creatine kinase	125	U/L	59-248
Blood urea nitrogen	8.2	mg/dL	8.0-20.0
Creatinine	0.88	mg/dL	0.65-1.07
K	4	mEq/L	3.6-4.8
Cl	107	mEq/L	101-108
C-reactive protein	0.06	mg/dL	≦0.14
Prothrombin time (INR)	0.91		0.80-1.20
aPTT	27.9	sec	25.4-36.9
D-dimer	3.3	μg/mL	≦1.0
Protein C activity	102	％	64-135
Protein S activity	74	％	64-149
Lupus anticoagulant	1.2	ratio	≦1.16
Anticardiolipin antibody	＜0.7	U/mL	≦12.3
Carcinoembryonic antigen	3	ng/mL	≦5
Carbohydrate antigen 19-9	28.7	U/mL	≦37
Urine blood	1+		-

**Figure 1:**
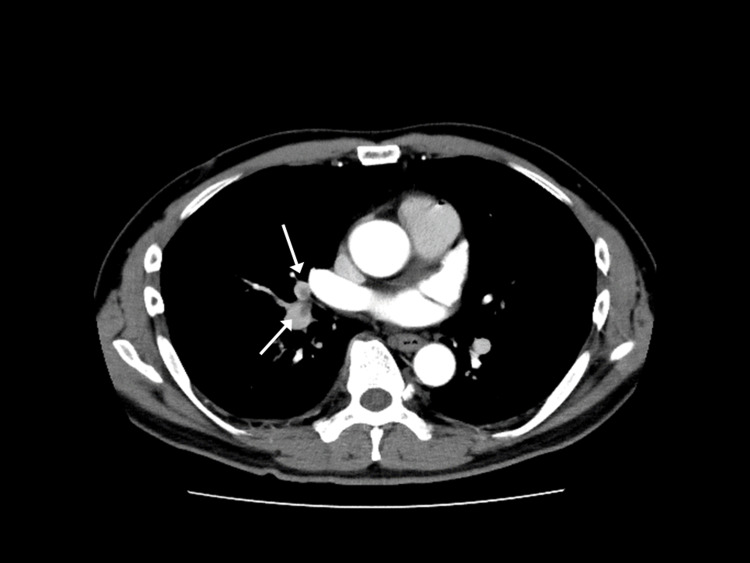
Pulmonary artery with a contrast defect (arrow).

**Figure 2:**
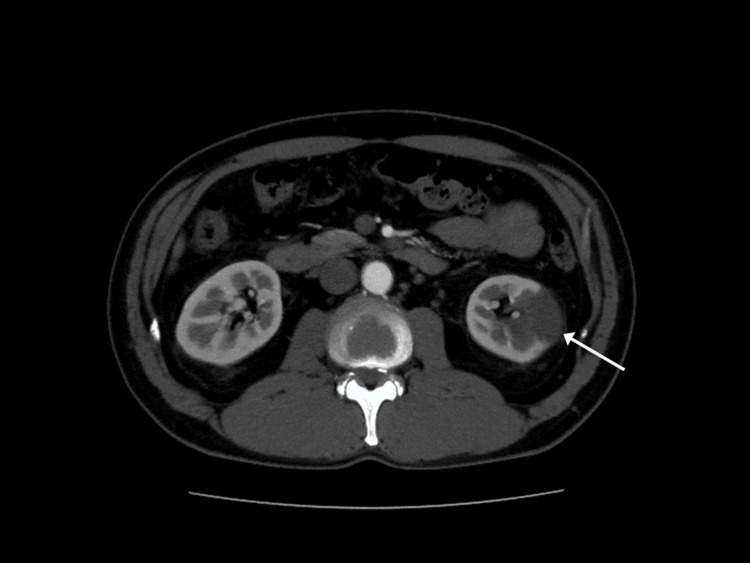
Part of the left kidney shows a contrast defect area (arrow).

Contrast-enhanced thoracoabdominal CT revealed thrombi in the bilateral pulmonary arteries and left renal hypodensity (Figures 1, 2).

There was no evidence of right ventricular enlargement on the CT.

**Figure 3:**
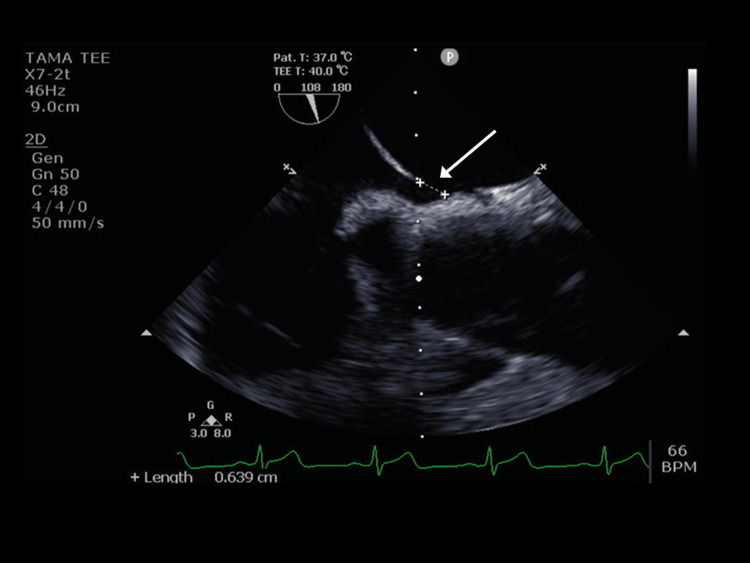
Transesophageal echocardiography reveals a 5-6-mm foramen ovale (arrow).

**Figure 4:**
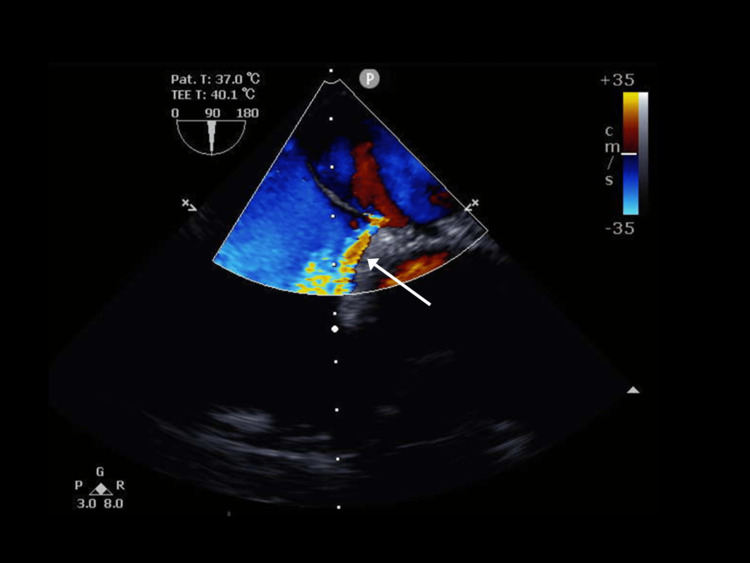
Transesophageal echocardiography shows blood flow from the left atrium to the right atrium at rest through the foramen ovale (arrow).

The patient was diagnosed with a pulmonary embolism and left renal infarction and was admitted to the Department of Cardiology. Anticoagulation therapy with heparin was started on the first day, and the back pain, which was scored on a numerical rating scale, improved. Transthoracic echocardiography (TTE) performed on the second day showed no valvular heart diseases, ASD, or PFO, indicating no evidence of pulmonary hypertension signs. A lower extremity venous ultrasound was also performed on the same day, which showed no thrombi in the bilateral femoral, popliteal, or inferior vena cava, and no findings were suggestive of deep vein thrombosis. Afterward, transesophageal echocardiography (TEE) performed on the sixth day revealed a 5-6 mm-diameter foramen ovale (Figures 3, 4).

**Figure 5:**
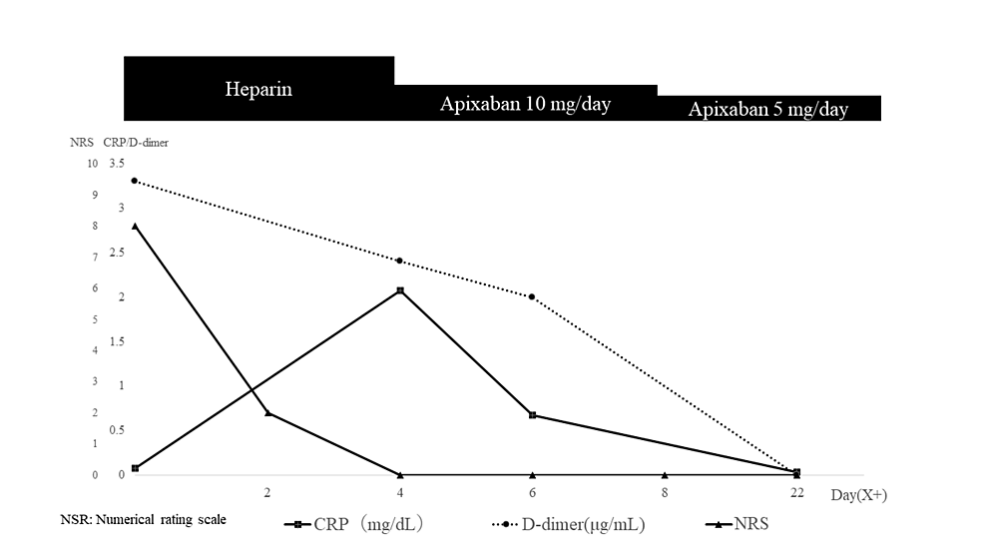
Clinical course

There was no thrombus in the left atrium or auricle, and the microbubble test was positive. On the fourth day, the patient was switched to the anticoagulant apixaban 10 mg. We performed a contrast-enhanced CT scan on the seventh day and found that the thrombus in the pulmonary artery had disappeared. In addition, blood tests showed no predisposition of thrombus formation, and the patient's pain had improved. We reduced the dose to 5 mg when the patient was discharged. The clinical course of the patient is shown in Figure 5.

T2-magnetic resonance imaging (MRI) of the head was performed at the outpatient clinic on the 14^th^ day after discharge and revealed a high-signal area in the left cerebellum. Magnetic resonance angiography showed no stenosis or occlusion of the vessel. Although asymptomatic, the patient was considered to have had a cryptogenic stroke due to the PFO and was transferred to a specialized hospital for catheter-based PFO closure.

## Discussion

A PFO is almost always asymptomatic but can present with symptoms related to its secondary complications, including cryptogenic strokes and Platypnea-Orthodeoxia syndrome [4], one of which is a paradoxical embolism.

In paradoxical embolisms, the thrombi that originate in the venous system migrate into the circulatory system via shunts, like an ASD, a PFO, and pulmonary arteriovenous fistulas, resulting in arterial embolisms. In the case of a PFO, the pressure range between the left and right atria is maintained, and the foramen ovale is closed under normal physiological conditions. However, when the right atrial pressure temporarily exceeds the left atrial pressure due to the Valsalva maneuver or a pulmonary embolism, a right-left shunt is formed, and embolic material formed in the systemic venous circulation is transported to the systemic arterial circulation, causing a paradoxical embolism [2].

The diagnosis of a paradoxical embolism requires 1) the absence of embolic material in the left ventricular system and the presence of an embolism in the systemic arterial circulation, 2) an embolic source in the venous system, and 3) an abnormal intracardiac or pulmonary arteriovenous pathway connecting the right and left ventricular systems [5]. In the present case, no deep vein thrombus was detected on lower extremity ultrasonography. This can be because the ultrasonography was performed after the start of anticoagulation therapy, and the remaining thrombus may have disappeared before the evaluation. Additionally, even though whole-leg ultrasonography has high power to detect deep vein thrombi in the lower extremities, it is extremely difficult and greatly depends on the skill of the operator [6]. Therefore, the possibility of a venous thrombus in the lower extremities cannot be ruled out with certainty.

Renal infarction can be caused by a multitude of cardiogenic reasons, such as atrial fibrillation, cardiomyopathies, artificial valves, endocarditis, and renal artery injuries, such as renal artery dissection, trauma, and hypercoagulable states such as a malignancy and antiphospholipid antibody syndrome [7]. However, none of these abnormalities were observed in this case. Therefore, the pathogenesis of this case was thought to be the formation of a deep venous thrombus in the lower extremities due to prolonged air travel. PE could have caused transient right to left shunt, enabling thrombus transit across PFO to arterial system.

A PubMed search using the keywords (Paradoxical embolism) (renal infarction) and (pulmonary embolism) identified only nine cases [8-16]. A summary of these cases is presented in Table 2.

**Table 2:** Reported cases of paradoxical renal embolism accompanied by pulmonary embolism PFO: Patent foramen ovale, IVC: Inferior vena cava, PH: Pulmonary hypertension VSD: Ventricular septal defect, DVT: Deep vein thrombosis, PE: Pulmonary embolism

Case	Age	Sex	Past medical history		Presentation			Sunt	Treatment			Outcome		References		
1	54	M	None mentioned		Altered mentation			PFO	Anticoagulation, IVC filter			Recovery		Ann Fr Anesth Réanim 2000;19:253-6 [8]		
2	47	F	None mentioned		Dyspnea, Chest pain, Left abdominal pain			PFO	Anticoagulation, IVC filter			Recovery		Am J Roentgenol 2004;183:1244-1246 [9]		
3	49	F	None		Dyspnea, Tachycardia, Left abdominal pain			PFO	Anticoagulation			Recovery		Nephrol Ther 2007;3:69-73 [10]		
4	52	M	Eisenmenger syndrome, Polycythemia vera, Heart failure, PH		Emesis, Left abdominal pain			VSD	Anticoagulation			Recovery		Intern Med 2021; 60: 3937-3940 [11]		
5	62	M	DM，CKD		Abdominal pain, Oligoanuria			PFO	Anticoagulation, Catheter-directed thrombolysis			Recovery		BMJ Case Rep 2022;15:e246885 [12]		
6	45	F	DVT		Abdominal pain, Emesis, Left lumbar pain, Dyspnea			PFO	Surgical removal of thrombus			Recovery		Am J Emerg Med. 1985;3:206-7 [13]		
7	61	M	Chest basal cell carcinoma		Back pain			PFO	Anticoagulaion			Recovery		J Cardiol Cases. 2018;19:19-21 [14]		
8	52	F	None		Dyspnea, Chest tightness			PFO	Anticoalugation			Recovery		Korean Circ J. 2012;42:853-6 [15]		
9	70	F	DM，Dyslipidemia, Hypertension, DVT, PE		Left lumbar pain			PFO	Anticoagulation, Vena cava filter, Catheter-directed thrombolysis			Recovery		Nephrol Dial Transplant. 2006;21:2315-7 [16]		

Six of the eight patients diagnosed with a paradoxical embolism due to a PFO were confirmed by TEE rather than TTE. Di Tullio et al. showed that TTE’s sensitivity and specificity for detecting PFOs were 28% and 100%, respectively [17]. The contrast TEE has a sensitivity of 89% and specificity of 100%, and the color Doppler method has a sensitivity of 100% and specificity of 100% [18]. Since TEE has a higher ability to detect intracardiac shunts, it should be performed in cases where an intracardiac shunt is suspected, even if TTE does not show an intracardiac shunt.

## Conclusions

In conclusion, when an arterial embolism of the systemic circulation is observed in a young patient with no underlying disease, it is important to investigate the possibility of paradoxical embolisms. In cases where the presence of an intracardiac shunt is suspected, it is essential to perform TEE to exclude it, even if TTE reveals no abnormalities.
